# Somatostatin receptor 2A protein expression characterizes anaplastic oligodendrogliomas with favorable outcome

**DOI:** 10.1186/s40478-018-0594-1

**Published:** 2018-09-07

**Authors:** Romain Appay, Emeline Tabouret, Mehdi Touat, Catherine Carpentier, Carole Colin, François Ducray, Ahmed Idbaih, Karima Mokhtari, Emmanuelle Uro-Coste, Caroline Dehais, Dominique Figarella-Branger, the POLA network, C. Desenclos, H. Sevestre, P. Menei, A. Rousseau, T. Cruel, S. Lopez, M.-I. Mihai, A. Petit, C. Adam, F. Parker, P. Dam-Hieu, I. Quintin-Roué, S. Eimer, H. Loiseau, L. Bekaert, F. Chapon, D. Ricard, C. Godfraind, T. Khallil, D. Cazals-Hatem, T. Faillot, C. Gaultier, M. C. Tortel, I. Carpiuc, P. Richard, W. Lahiani, H. Aubriot-Lorton, F. Ghiringhelli, C. A. Maurage, C. Ramirez, E. M. Gueye, F. Labrousse, O. Chinot, L. Bauchet, V. Rigau, P. Beauchesne, G. Gauchotte, M. Campone, D. Loussouarn, D. Fontaine, F. Vandenbos-Burel, A. Le Floch, P. Roger, C. Blechet, M. Fesneau, A. Carpentier, J. Y. Delattre, S. Elouadhani-Hamdi, M. Polivka, D. Larrieu-Ciron, S. Milin, P. Colin, M. D. Diebold, D. Chiforeanu, E. Vauleon, O. Langlois, A. Laquerriere, F. Forest, M. J. Motso-Fotso, M. Andraud, G. Runavot, B. Lhermitte, G. Noel, S. Gaillard, C. Villa, N. Desse, C. Rousselot-Denis, I. Zemmoura, E. Cohen-Moyal, E. Uro-Coste, F. Dhermain

**Affiliations:** 1 APHM, Hôpital de la Timone, Service d’Anatomie Pathologique et de Neuropathologie, Marseille, France; 2 APHM, Hôpital de la Timone, Service de Neurooncologie, Marseille, France; 3 Aix-Marseille Univ, CNRS, INP, Inst Neurophysiopathol, Marseille, France; 4 AP-HP, Hôpitaux Universitaires La Pitié Salpêtrière-Charles Foix, Service de Neurologie 2–Mazarin, Paris, France; 5 Sorbonne Université, Inserm, CNRS, UMR S 1127, Institut du Cerveau et de la Moelle épinière, ICM, AP-HP, Hôpitaux Universitaires La Pitié Salpêtrière - Charles Foix, Service de Neurologie 2-Mazarin, F-75013 Paris, France; 6 Hospices Civils de Lyon, Hôpital Pierre Wertheimer, Service de Neuro-oncologie, Bron, France; 7 Department of Cancer Cell Plasticity, Cancer Research Centre of Lyon, Inserm U1052, CNRS UMR5286, Lyon, France; 8 AP-HP, Hôpitaux Universitaires La Pitié Salpêtrière-Charles Foix, Service de Neuropathologie Raymond Escourolle, Paris, France; 9 CHU Toulouse, Hôpital Rangueil, Service d’Anatomie Pathologique et Histologie-Cytologie, Toulouse, France; 10 Inserm U1037, Centre de Recherche en Cancérologie de Toulouse, Université de Toulouse, Toulouse, France

**Keywords:** Somatostatin receptor subtype 2A (SSTR2A), Glioma, Biomarker, Therapeutic target

## Abstract

Diffuse gliomas are classified according to the 2016 WHO Classification of Tumors of the Central Nervous System, which now defines entities by both histology and molecular features. Somatostatin receptor subtype 2A (SSTR2A) expression has been reported in various solid tumors as associated with favorable outcomes. Its expression has been reported in gliomas with uncertain results regarding its prognostic value. The objective of this study was to assess the prognostic impact of SSTR2A protein expression in a large cohort of grade III and IV gliomas classified according to the updated 2016 WHO classification. We further validated our result with an independent cohort of low grade glioma using dataset generated by The Cancer Genome Atlas (TCGA) Research Network.

We analyzed clinical and molecular data from 575 patients. SSTR2A protein expression was evaluated using immunohistochemistry on tissue microarrays. High expression of SSTR2A protein associated with the anaplastic oligodendroglioma *IDH*-mutant and 1p/19q-codeleted subgroup (*p* < 0.001). Among these tumors, SSTR2A protein expression was significantly associated with a lower proliferative index, the absence of microvascular proliferation and the absence of necrosis (*p* < 0.001). Furthermore SSTR2A protein expression associated with better overall survival (*p* = 0.007) and progression-free survival (*p* = 0.01) in both univariate and multivariate analysis when adjusted by the age, the presence of necrosis and the mitotic index. Similar results were obtained regarding SSTR2 mRNA expression in the TCGA low grade glioma, subtype *IDH*-mutant and 1p/19q-codeleted, dataset.

SSTR2A might represent an attractive biomarker and therapeutic target in anaplastic oligodendroglioma *IDH*-mutant and 1p/19q-codeleted specific subgroup. Understanding the implicated molecular pathways may represent a step forward to improve therapeutic approaches.

## Introduction

Diffuse gliomas are the most common primary brain cancers. They are classified according to the 2016 WHO (World Health Organization) Classification of Tumors of the Central Nervous System (CNS), which combines for the first time histological and molecular features in an “integrated diagnosis” [20]. This novel classification system incorporates mutations in the isocitrate deshydrogenase 1 and 2 genes (*IDH1* and *IDH2*) and the whole-arm chromosomal loss of 1p and 19q (1p/19q-codeletion), which are both required to be present for confirming the diagnosis of oligodendroglioma. *IDH* mutations are the key genetic alterations characterizing grade II and III gliomas and glioblastomas with favorable outcome [37]. Diagnostic strategy and therapeutic management depend on each subtype and the identification of distinct prognostic subgroups among gliomas belonging to the same histo-molecular category is crucial to open perspectives of therapeutic development.

Somatostatin (SST), also known as growth hormone-inhibiting hormone (GHIH), was first described in 1968 as a hormone secretion [18]. The effects of SST are mediated through its interaction with somatostatin receptors (SSTR), a family of G protein-coupled receptors consisting of 6 different subtypes (SSTR1, 2A, 2B, 3, 4 and 5) [26, 32].

SSTR2A is the predominant subtype. Its expression has been reported in various solid tumors as associated with favorable outcomes [1, 19, 23, 25, 28]. SSTRs are commonly expressed on neuroendocrine tumors (NETs). In NETs, the expression of SSTR2A by tumor cells is of interest for both diagnostic and therapeutic strategy. Indeed, SSTR2A is a target for radiolabeled imaging (OCTREOSCAN, PET 68Ga-DOTATOC) as well as therapy using SST analogs labelled with β-emitting isotopes (90Y-DOTATOC and 177Lu-DOTATATE) [2, 5, 29]. In addition, SST analogs (Octreotide and Lanreotide) are used to inhibit the release of hormones and control secretory symptoms [1, 13, 14, 16, 26]. Interestingly, recent studies demonstrated that SST analogs can also inhibit growth of SSTRs-dependent tumors by regulating intracellular signaling pathways, including dephosphorylation of actors implicated in the mitogen-activated protein kinase (MAPK) signaling and induction of apoptosis [13, 26, 32].

Few studies have previously reported the expression of SSTR2A in gliomas with discrepant results regarding their association with grade [11, 17, 21, 26]. In a recent study, Kiviniemi et al. [17] reported high expression of SSTR2A protein predominant in oligodendrogliomas in a cohort of 184 gliomas classified according to the specific molecular signatures of the updated WHO classification. Furthermore, they reported a survival benefit in gliomas with high expression of SSTR2A protein. However, this difference might be related to the association between SSTR2A and the oligodendroglioma subtype and it is not clear whether the level of SSTR2A expression has prognostic significance among the oligodendroglioma subgroup.

In France, since 2008, the POLA network provides a centralized review and molecular analysis of de novo adult high-grade glioma with an oligodendroglial component. Using the tissue samples and dataset provided by this network, our objective was to assess the prognostic impact of the SSTR2A protein expression in a large cohort of grade III and IV gliomas. We further validated our result with an independent cohort using dataset generated by the TCGA Research Network [8].

## Materials and methods

### Study population

A total number of 575 patients from the French nation-wide POLA cohort were included in this study. Inclusion criteria were the written consent of the patient for clinical data collection and genetic analysis according to national and POLA network policies, sufficient tissue material for molecular studies allowing classification according to the WHO 2016 (i.e. evaluation of the *IDH* mutation and 1p/19q-codeletion status) and an established diagnosis of high grade glioma (WHO grade III or IV).

*IDH* mutation status was evaluated using automated immunohistochemistry (IHC) and direct sequencing using the Sanger method as previously described [30]. The genomic profile and assessment of the 1p/19q-codeletion status was determined based on single nucleotide polymorphism (SNP) arrays, comparative genomic hybridization (CGH) arrays, or microsatellite marker analysis as previously described [30].

Anaplastic oligodendroglioma, *IDH*-mutant, 1p19q-codeleted were classified into 3 pathological subgroups based on mitotic index, microvascular proliferation (MVP), and necrosis [12]. Group 1, involved cases with more than 5 mitoses per 10-high power field (HPF), no MVP, and no necrosis, group 2 displayed MVP but no necrosis, and group 3 showed MVP and necrosis.

Proliferative index was evaluated using Ki67 antibody (clone Mib1; 1:100; Dako) and scored as percentage by counting the immunostained nuclei of 400 cells in the most positive area.

Clinical data collected included gender, age at surgery, extend of surgical resection, preoperative Karnofsky performance scale (KPS) and post-operative treatment. Clinical information regarding the outcome was recorded as follow: progression-free survival (PFS) was defined as the time from the date of surgery to recurrence or death from any cause, censored at the date of the last documented disease evaluation. Overall survival (OS) was defined as the time from the date of surgery to death from any cause, censored at the date of last contact.

### Tissue microarray (TMA) design

Tissue microarrays (TMA) were constructed from routinely processed formalin-fixed paraffin-embedded tumor material. Areas of viable and representative tumor, away from foci of necrosis and from grade II areas when present, following review of all blocks were marked by a pathologist (DFB) prior to inclusion into the TMA. In order to accurately study tumor heterogeneity, for each case three replicate 0.6 mm cores were sampled from different tumor areas.

### SSTR2A immunohistochemistry and evaluation on TMA

Immunohistochemical detection of SSTR2A protein expression was performed on 5 μm thick TMA sections with Ventana Benchmark XT. The monoclonal SSTR2A antibody (clone UMB1) was purchased from Abcam and used at 1/4000 dilution. A Benchmark Ventana autostainer (Ventana Medical Systems SA, Illkirch, France) was used for detection and TMA slides were simultaneously immunostained to avoid inter-manipulation variability.

Immunostaining was scored for 185 triplicates by two independent pathologists (RA & DFB). In almost all cases the staining intensity was roughly similar among the different cores of the triplicate, thus the core demonstrating the stronger immunoreaction was analyzed to establish an immunoreactive score (IRS) as previously described by Casar-Borota et al. [9]. Staining intensity was scored as 0 (no immunostaining), 1 (weak), 2 (moderate), or 3 (strong). The percentage of immunoreactive cells was scored as 0 (none), 1 (< 10%), 2 (10–50%), 3 (51–80%), or 4 (> 80%). Multiplication of the staining intensity score and the percent immunoreactivity score resulted in an IRS score, which ranged from 0 to 12. Because the results were highly concordant among the two pathologists, the 390 remaining triplicates were scored by a single pathologist (RA).

**Fig. 1:**
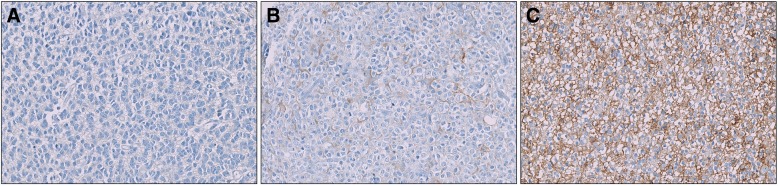
Immunohistochemical staining of SSTR2A protein in anaplastic oligodendroglioma *IDH*-mutant and 1p/19q-codeleted **(a)** Negative staining (IRS = 0) **(b)** Weak staining intensity (score = 1) in 20% of the cells (score = 2) corresponding to positive cases with low expression (IRS = 3) **(c)** High staining intensity (score = 3) in 100% of the cells (score = 4) corresponding to positive cases with high expression (IRS = 12)

The median IRS score based on analysis of all cases in the cohort was equal to 1 and corresponded to a cutoff between positive (IRS ≥ 1) and negative expression (IRS = 0). The third quartile (IRS = 4) was used as the cutoff to define positive cases with high expression (IRS ≥ 4) versus positive cases with low expression (1 ≤ IRS < 4). Representative images of different IRS are shown in Fig. 1.

### Public data acquisition

Informed consent and ethical approval was obtained when The National Cancer Institute (NCI) and National Human Genome Research Institute (NHGRI) had collected tissue for TCGA [8]. All sequencing or clinical datasets used in the present study are publicly available to researchers without restrictions or limitations according to TCGA policy. Therefore, the results shown here are in whole based upon data generated by the TCGA Research Network: http://cancergenome.nih.gov/. We used GlioVis data portal for visualization and analysis of brain tumor expression datasets [6] (http://gliovis.bioinfo.cnio.es/), we limited our analysis to data from adult patients with LGG.

### Statistical analysis

For statistical analysis, the Chi-square test was used to compare qualitative variables when they were scored as positive or negative. Continuous variables were compared using the Mann–Whitney U test. The Kaplan–Meier method was used to estimate survival distributions. Log-rank tests were used for univariate comparisons. Age at diagnosis, KPS, presence of necrosis and proliferative index were used to build the multivariate Cox proportional hazard backward models. All statistical tests were two-sided, and the threshold for statistical significance was *p* = 0.05. Statistical analysis was done using IBM SPSS statistics software version 23 (IBM SPSS Inc., Chicago, IL, USA).

## Results

### Clinicopathologic characteristics

We evaluated 227 anaplastic oligodendroglioma *IDH*-mutant and 1p/19q-codeleted. In addition, we analyzed 86 anaplastic astrocytoma (80 *IDH*-mutant and 6 *IDH*-wildtype) and 262 glioblastomas (124 *IDH*-mutant and 138 *IDH*-wildtype). Overall 575 patients were included in this study. Patient characteristics are presented in Table 1.

**Table 1:** Characteristics of the patient population (*N* = 583)

	Anaplastic astrocytoma IDH-wildtype *N* (% of Total)	Glioblastoma IDH-wildtype *N* (% of Total)	Anaplastic astrocytoma IDH-mutant *N* (% of Total)	Glioblastoma IDH-mutant *N* (% of Total)	Anaplastic oligodendroglioma IDH-mutant and 1p/19q-codeleted *N* (% of Total)
Total	6	138	80	124	227
Median age, years (range)	52 (26–60)	59 (16–83)	37 (18–75)	38 (19–78)	48 (19–80)
Gender					
Female	2 (33)	60 (44)	35 (44)	53 (43)	101 (44)
Male	4 (67)	78 (56)	45 (56)	71 (57)	126 (56)
Median preoperative KPS% (range)	90 (90–100)	80 (40–100)	90 (60–100)	90 (50–100)	90 (50–100)
Resection					
Gross total resection	1 (17)	57 (41)	18 (23)	34 (27)	81 (36)
Subtotal resection	2 (33)	37 (27)	38 (48)	43 (35)	58 (26)
Biopsy	2 (33)	25 (18)	9 (11)	35 (28)	66 (29)
Unknown	1 (17)	19 (14)	15 (19)	12 (10)	22 (10)
Adjuvant treatment					
None	0	3 (2)	3 (4)	1 (1)	9 (4)
RT alone	1 (17)	3 (2)	5 (6)	3 (2)	42 (19)
RT + PCV	0	2 (1)	15 (19)	16 (13)	72 (32)
PCV alone	0	2 (1)	0	0	6 (3)
RT + TMZ	5 (83)	104 (75)	38 (48)	73 (59)	70 (31)
TMZ alone	0	5 (4)	3 (4)	4 (3)	5 (2)
No data	0	19 (14)	16 (20)	27	23 (10)
IRS Score					
IRS = 0	5 (83)	86 (62)	31 (39)	47 (38)	69 (30)
1 ≥ IRS < 4	0	33 (24)	27 (34)	46 (37)	51 (22)
IRS ≥ 4	1 (17)	19 (14)	22 (27)	31 (25)	107 (47)

Abbreviations: *IRS* Immunoreactive score; *KPS* Karnofsky Performance Status Scale; *PCV* Procarbazine + Lomustine + Vincristine; *RT* Radiotherapy; *TMZ* Temozolomide

### Scoring of SSTR2A immunohistochemistry and its association with tumor entity

**Fig. 2:**
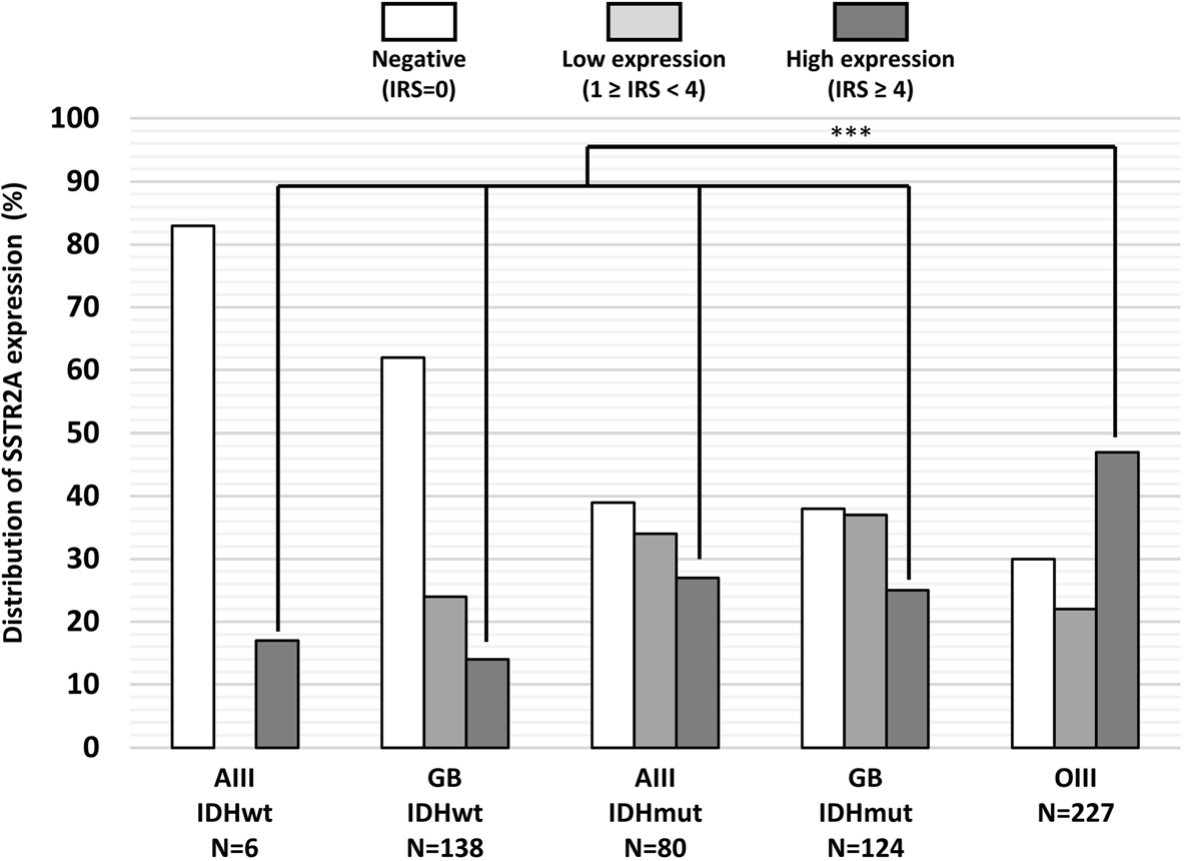
Distribution of SSTR2A protein expression according to tumor subtype Abbreviations: AIII IDHwt, Anaplastic astrocytoma *IDH*-wildtype; GB IDHwt, Glioblastoma *IDH*-wildtype; AIII IDHmut, Anaplastic astrocytoma *IDH*-mutant; GB IDHmut, Glioblastoma *IDH*-mutant; OIII, Anaplastic oligodendroglioma, *IDH*-mutant and 1p/19q-codeleted. *** = *p* < 0.001

Expression (any level; IRS ≥ 1) of SSTR2A was detected in 59% (337/575) of gliomas. The distribution of SSTR2A protein expression according to gliomas subtype is shown in Fig. 2. SSTR2A protein expression was significantly associated with *IDH* mutation (66% of *IDH*-mutant tumors were positive for SSTR2A expression versus 39% of *IDH*-wild type, *p* < 0.001). High expression of SSTR2A (IRS score ≥ 4) was detected in 31% (180/575) of gliomas. High expression of SSTR2A was significantly associated with anaplastic oligodendroglioma, *IDH*-mutant and 1p/19q-codeleted and was found in approximatively half of the studied samples whereas it was uncommon in astrocytoma and glioblastoma independently of the presence of *IDH*-mutation (*p* < 0.001).

### Association between expression of SSTR2A protein and the anaplastic oligodendroglioma *IDH*-mutant and 1p/19q-codeleted pathological subgroups

**Fig. 3:**
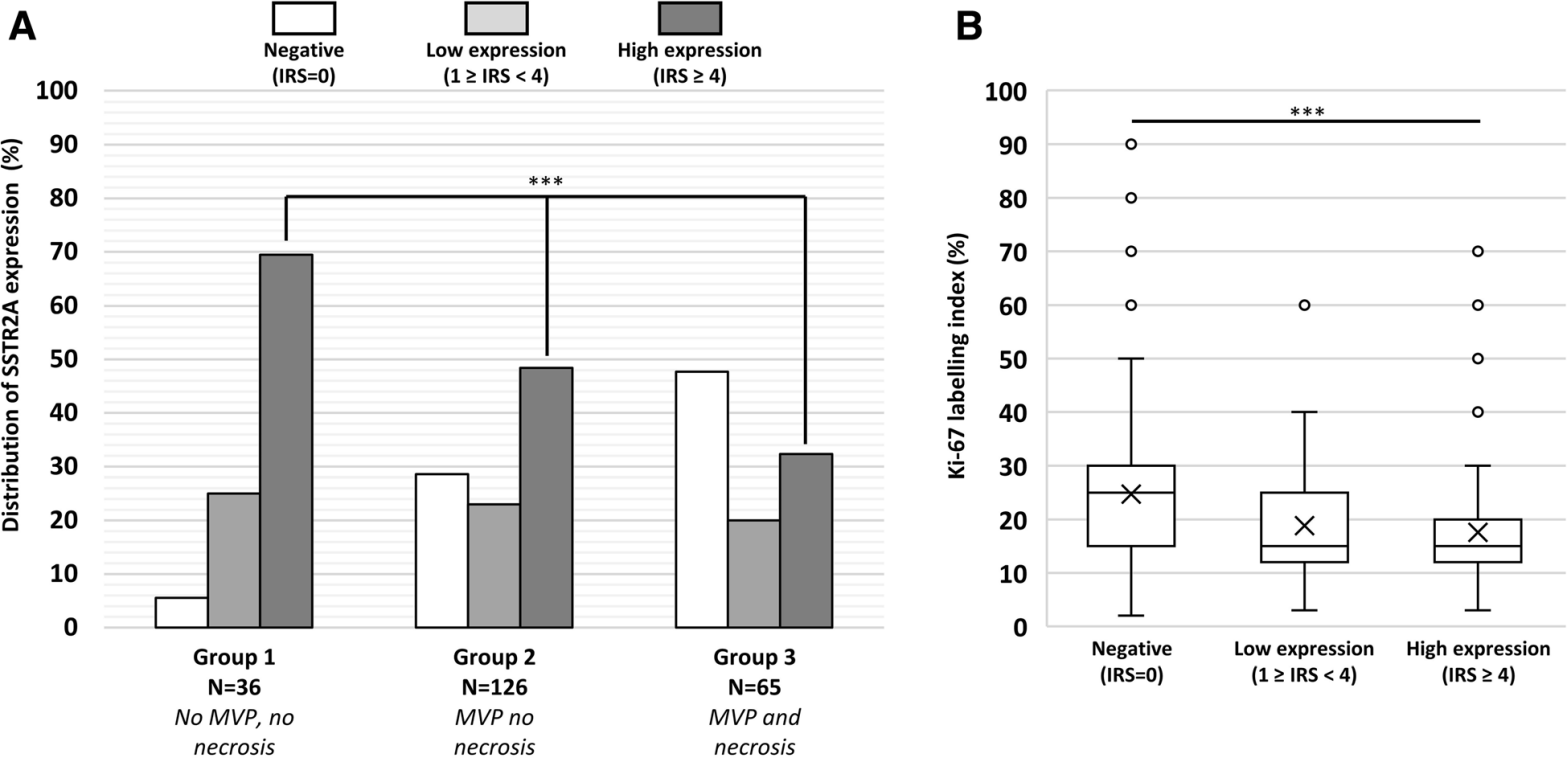
**a** Distribution of SSTR2A protein expression according to pathological groups in anaplastic oligodendrogliomas, *IDH*-mutant and 1p/19q-codeleted. Abbreviation: MVP, microvascular proliferation **b** Ki-67 labelling index according to SSTR2A protein expression among the anaplastic oligodendroglioma, *IDH*-mutant and 1p/19q-codeleted. *** = *p* < 0.001

In the anaplastic oligodendroglioma subgroup, expression levels of SSTR2A protein varied depending on the pathological subgroup as shown in Fig. 3a (*p* < 0.001). High SSTR2A expression was detected in the majority (70%) of anaplastic oligodendroglioma belonging to the pathological group 1. It was less frequent in group 2 (50%) and only one-third of the anaplastic oligodendroglioma of group 3 presented with high SSTR2A expression. In accordance with the previous result, the absence of SSTR2A expression was uncommon among the patients of the group 1 (5%) whereas approximately half of the group 3 presented with no SSTR2A expression.

We selected six representative cases (i.e. two negative for SSTR2A expression, IRS = 0; two positive with low expression, IRS = 3; and two positive with high expression, IRS = 9) from anaplastic oligodendroglioma, *IDH*-mutant and 1p/19q-codeleted belonging to the group 3 and evaluated intratumoral heterogeneity of SSTR2A expression on whole tumor sample. For each selected case, the percentage and intensity of the immunostaining was in agreement with the result obtained on TMA. One of the two cases evaluated with an IRS score equal to 0 showed focal positivity away from the area selected for the TMA (this area corresponded to a much less aggressive part of the tumor) but the vast majority of the sample was negative, the second one was completely negative. In the other four cases, the SSTR2A protein expression was in keeping with results evaluated on TMA and always absent in tumor cells located in the vicinity of foci of palisading necrosis (data not shown). Therefore, the low SSTR2A expression in group 3 is not related to necrosis per se but likely represent the acquisition of a more aggressive phenotype. It should be noted that SSTR2A protein expression was also observed in neurons within the peripheral cortex.

### Association between expression of SSTR2A protein and proliferative index in anaplastic oligodendroglioma, IDH-mutant and 1p/19q-codeleted

To examine whether SSTR2A protein expression was associated with differences in the proliferative index we compared quantitative evaluation of Ki-67 (MIB-1) labelling index using immunohistochemistry. As presented in Fig. 3b, anaplastic oligodendroglioma, *IDH*-mutant and 1p/19q-codeleted with high or low SSTR2A protein expression presented with a lower Ki-67 labelling index when compared to SSTR2A negative gliomas (median Ki-67 expression = 15% in SSTR2A positive gliomas versus 26% in SSTR2A negative, *p* < 0.001).

### Association between SSTR2A protein expression and survival in anaplastic oligodendroglioma, *IDH*-mutant and 1p/19q-codeleted

**Fig. 4:**
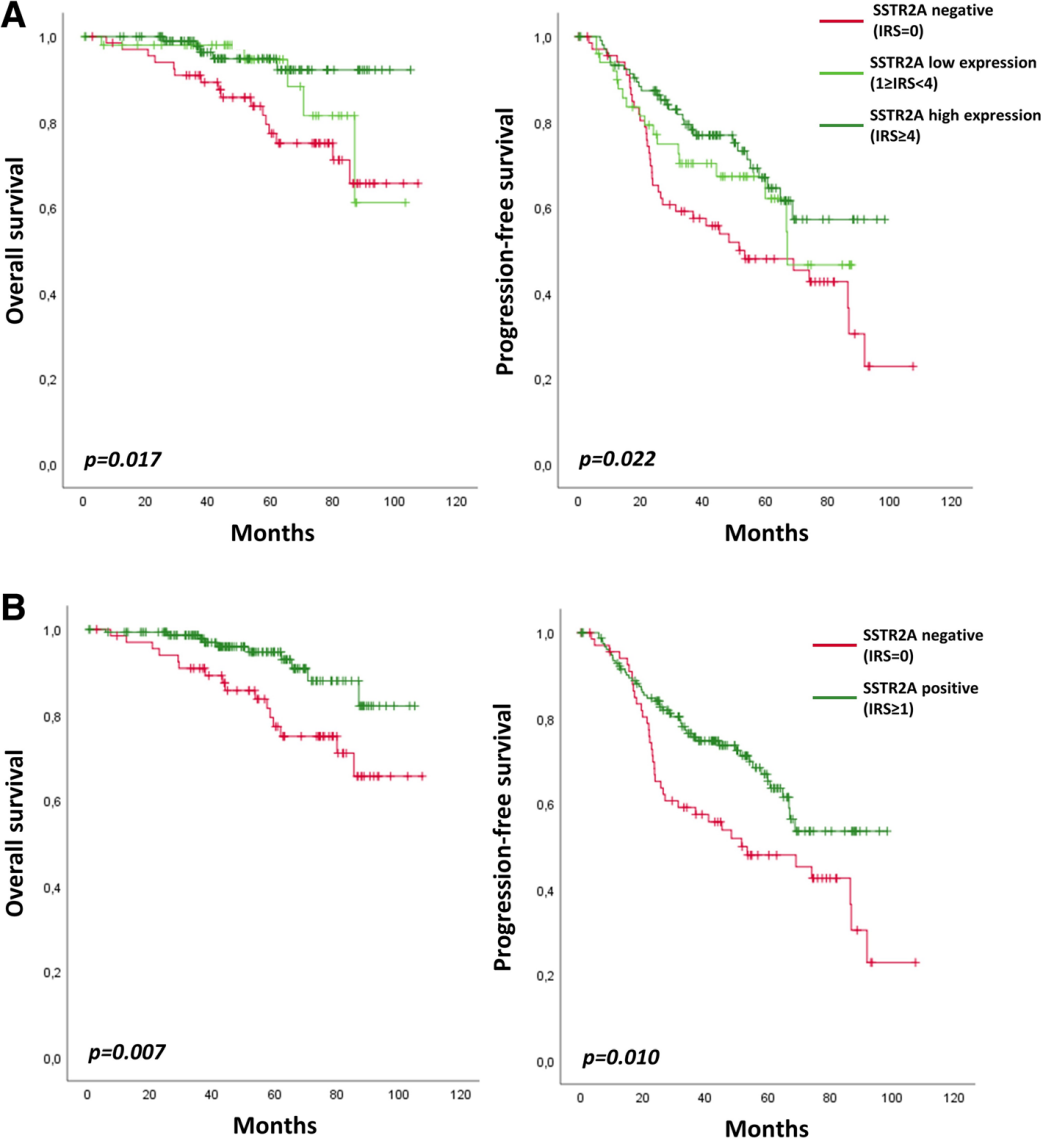
Overall survival and Progression-free survival according to SSTR2A protein expression in anaplastic oligodendroglioma, *IDH*-mutant and 1p/19q-codeleted. **a** No SSTR2A expression (IRS = 0) versus low SSTR2A expression (1 ≤ IRS < 4) versus high SSTR2A expression (IRS ≥ 4). **b** Negative (IRS = 0) versus positive (IRS ≥ 1) SSTR2A expression

We further analyzed the prognostic significance of SSTR2A expression in gliomas. Among the anaplastic oligodendroglioma, *IDH*-mutant and 1p/19q-codeleted, SSTR2A protein expression is prognostic for PFS and OS (Fig. 4a). Both low and high SSTR2A expressive anaplastic oligodendroglioma presented with better OS (*p* = 0.022) and PFS (*p* = 0.017) when compared to negative gliomas. No significant prognostic difference was observed between low expression and high expression in terms of PFS (*p* = 0.293) and OS (*p* = 0.280).

Accordingly, expression of SSTR2A protein (any level, IRS ≥ 1) was significantly associated with longer PFS (*p* = 0.010) and OS (*p* = 0.007) among the subgroup of anaplastic oligodendroglioma, IDH-mutant and 1p/19q-codeleted (Fig. 4b).

In multivariate analysis, expression of SSTR2A (any level, IRS ≥ 1) was also significantly associated with better OS when adjusted by the age (HR = 0.414; 95% CI, 0.185–0.929; *p* = 0.033), by the presence of necrosis (HR = 0.391; 95% CI, 0.174–0,877; *p* = 0.023) or by the proliferative index (HR = 0.411; 95% CI, 0.176–0.959; *p* = 0.04). When adjusted by the preoperative KPS, the result did not reach statistical significance (HR = 0.413; 95% CI, 0.165–1.034; *p* = 0.059), which might be attributed to an insufficient number of collected data. The extend of surgical resection and postoperative treatment did not reached a *p*-value > 0.2 in univariate analysis thus have not been integrated in the multivariate analysis.

No association between survival and SSTR2A protein expression was observed in patients with other gliomas subtypes (data not shown).

### TCGA low-grade glioma RNA-seq data as confirmation dataset

**Fig. 5:**
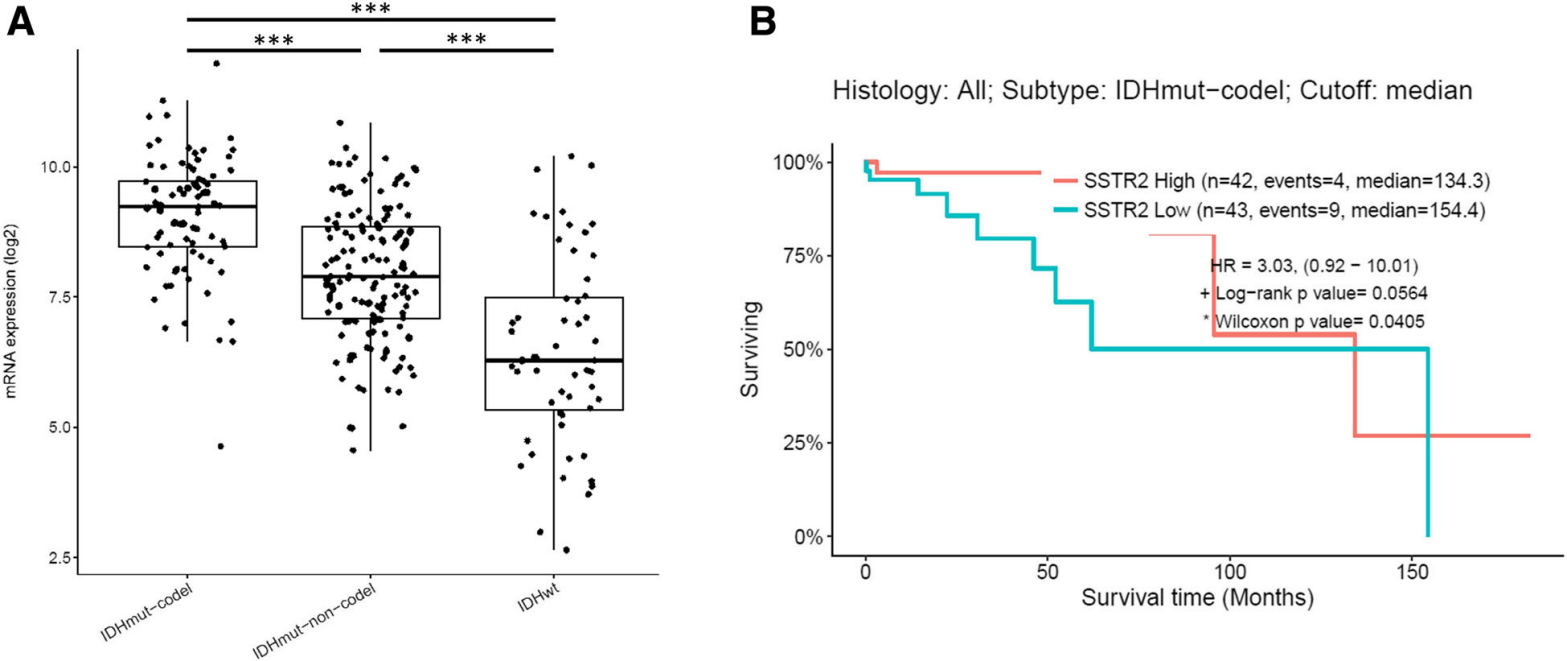
**a** Relation between SSTR2 mRNA expression and subtype in low grade glioma patients from TCGA cohort. **b** Overall survival according to SSTR2 mRNA expression in patients with *IDH*-mutant and 1p/19q-codeleted low grade gliomas from the TCGA cohort. *** = *p* < 0.001

As presented in Fig. 5a, among the independent TCGA low-grade glioma dataset we observed a higher SSTR2 mRNA expression in *IDH*-mutant glioma when compared to *IDH*-wild type (*p* < 0.001). Furthermore, *IDH*-mutant and 1p/19q-codeleted gliomas presented with the highest level of SSTR2 mRNA expression (*p* < 0.001).

When categorized into two groups according to the median score, we observed a better overall survival in glioma with high SSTR2 mRNA expression (*p* = 0.056) among the low grade glioma *IDH*-mutant and 1p/19q-codeleted subgroup (Fig. 5b).

No association between survival and SSTR2 mRNA expression was observed in patient with *IDH*-mutant without 1p/19q-codeletion (*p* = 0.478) and *IDH*-wildtype gliomas (*p* = 0.301) (data not shown).

## Discussion

Gliomas are the most common primary CNS tumors. Updated WHO classification of CNS tumors combines for the first time histological and molecular features for an integrated diagnosis. *IDH*-mutant gliomas display a more favorable outcome than the *IDH*-wildtype counterpart. However, despite aggressive treatment, *IDH*-mutant gliomas are characterized by a malignant transformation over time with a median survival of approximately 10 years. Therefore, the identification of distinct prognostic groups among *IDH*-mutant gliomas might be of interest to better stratified the patients and improve therapeutic approaches.

In the present study, we have evaluated the protein expression of SSTR2A by immunohistochemistry in a large cohort of gliomas classified according to the WHO 2016 classification. Because of the inclusion criteria defined by the POLA network (i.e. high-grade glioma with oligodendroglial component) it is worth noticing that the percentage of each category in our study does not reflect the normal distribution of gliomas.

In our cohort, most *IDH*-wild type gliomas did not express SSTR2A protein and a significant overexpression of SSTR2A protein was observed in the *IDH*-mutant gliomas. Among these, the highest expression was recorded in the anaplastic oligodendroglioma, *IDH*-mutant and 1p/19q-codeleted subgroup, which is consistent with previous observations [17]. Moreover, in these tumors, SSTR2A protein expression was associated with a lower proliferative index, the absence of microvascular proliferation and the absence of necrosis (group 1) while it is less expressed in group 2 and 3. Of interest, in anaplastic oligodendroglioma *IDH*-mutant and 1p/19q-codeleted, we observed a significant association between expression of SSTR2A protein and favorable outcome (as indicated by longer PFS and OS in this subgroup of tumors expressing SSTR2A). Importantly, association between SSTR2A expression and outcome remained significant in multivariate analysis adjusting for known prognostic factors in this subtype. Furthermore, similar results were obtained regarding SSTR2 mRNA expression in an independent cohort using the low grade gliomas TCGA dataset.

Thus, our results indicate that the immunohistochemical expression of SSTR2A protein could serve as a prognostic biomarker among anaplastic oligodendroglioma, *IDH*-mutant and 1p/19q-codeleted.

SSTR2A is strongly expressed in neuroendocrine tumors and also in normal neurons according to the brain transcriptome database [7]. The high expression of SSTR2A in *IDH-*mutant adult high grade gliomas in comparison to *IDH*-wildtype gliomas is in accordance with the proneural subtype of *IDH*-mutant gliomas reported by the Cancer genome Atlas [33]. Importantly, among this group, the highest SSTR2A expression is recorded in anaplastic oligodendrogliomas *IDH*-mutant and 1p/19q-codeleted. This is in keeping with the neuronal differentiation of these tumors highlighted by ultrastructural and transcriptional studies [4, 10, 35]. Indeed, Ducray et al. [10] demonstrated that there is a strong correlation between 1p/19q-codeletion and the expression of proneural genes in malignant gliomas. Interestingly, in their cohort, SSTR2 was significantly overexpressed (*p* = 0.0001) in the 1p/19q-codeleted group when compared to the EGFR amplified high grade gliomas. Furthermore, Bielle et al. [4] reported the occurrence of neuronal intermediate progenitors (NIP) markers in a subset of anaplastic oligodendroglioma *IDH*-mutant and 1p/19q-codeleted especially in cases associated with necrosis (*p* = 0.0034). It is possible that NIP-high subgroup could result from tumor dedifferentiation. In the same line, we can postulate that loss of SSTR2A expression among anaplastic oligodendrogliomas *IDH*-mutant and 1p/19q-codeleted is correlated to the acquisition of a more immature neuronal phenotype. We could hypothesize that SSTR2A, through its inhibition of the MAPK-pathway and induction of apoptosis, may constitute a brake against in the acquisition of markers of aggressiveness.

Surgery, when possible, is the first line treatment for *IDH*-mutant and 1p/19q-codeleted oligodendroglioma and “watch-and-wait” strategies may be justified in patients with macroscopically complete resection or in young patients (aged < 40 years) with incomplete resection if the tumor is not symptomatic [36]. If further treatment beyond surgery is considered necessary the standard treatment is radiotherapy followed by procarbazine, lomustine and vincristine chemotherapy (PCV). “Watch-and-wait” strategies might be considered even with WHO grade III tumors [36]. According to our results, SSTR2A protein expression is an independent biomarker of good prognosis in both uni- and multivariate analysis that might guide the decision of a postsurgical treatment among the subgroup of anaplastic oligodendrogliomas, *IDH*-mutant and 1p/19q-codeleted.

Moreover, SSTR2A is an attractive therapeutic target. An inhibitory effect of SST analogs on cell migration and invasion has been demonstrated in various tumors [3, 24, 31, 34]. Moreover, therapy using radiolabeled SST analogs is commonly used as a therapeutic option for the treatment of metastatic or inoperable neuroendocrine tumors [5, 29]. Interestingly, encouraging results have been reported in some studies evaluating locoregional delivery of targeted radionuclide therapy in gliomas [15, 22, 27]. Our results suggest the identification of the specific oligodendroglioma subgroup as a possible target of therapies using SST analogs.

## Conclusion

Our results demonstrate that high expression of SSTR2A is commonly associated with anaplastic oligodendrogliomas. Moreover, among the anaplastic oligodendroglioma subgroup SSTR2A expression is associated with a better OS. Therefore, SSTR2A might represent an attractive biomarker and target for these tumors.

## Abbreviations

CNS: Central Nervous System;
HPF: High power field (HPF)
IDH 1 & 2: Isocitrate deshydrogenase 1 and 2 genes
IRS: Immunoreactive score
KPS: Karnofsky performance status scale
MVP: Microvascular proliferation
NIP: Neuronal Intermediate Progenitor
OS: Overall survival
PCV: Procarbazine + Lomustine + Vincristine chemotherapy
PFS: Progression-free survival
RT: Radiotherapy
SST: Somatostatin
SSTR2A: Somatostatin receptor subtype 2A
TCGA: The Cancer Genome Atlas
TMA: Tissue microarray
TMZ: Temozolomide
